# Chilaiditi Sign: A Rare Radiographic Encounter and Diagnostic Exploration

**DOI:** 10.7759/cureus.57822

**Published:** 2024-04-08

**Authors:** Souvik Sarkar, Sandeep Reddy Ramala

**Affiliations:** 1 Respiratory Medicine, Datta Meghe Institute of Higher Education and Research, Wardha, IND; 2 General Surgery, Jawaharlal Nehru Medical College, Datta Meghe Institute of Higher Education and Research, Wardha, IND

**Keywords:** pneumoperitoneum, total abdominal hysterectomy, uterine mass, chilaiditi’s syndrome, chilaiditi sign

## Abstract

This case report explores the complex diagnostic dilemma between the Chilaiditi sign and the pneumoperitoneum. The patient presented with chronic complaints of vague abdominal pain, abdominal distension, and breathlessness. A chest X-ray indicated an elevated right hemidiaphragm with transverse colon interposition, leading to the diagnosis of Chilaiditi's sign. Subsequent imaging, including abdominal ultrasound and contrast-enhanced computed tomography, revealed a large non-enhancing multilobulated multicystic mass adherent to the anterior wall of the uterus, raising suspicions of malignancy. Managed by gynecologists, the patient underwent a total abdominal hysterectomy, mass excision, and bilateral salpingo-oophorectomy. The discussion delves into Chilaiditi's sign, its historical context, and its complex pathophysiology involving intestinal, hepatic, and/or diaphragmatic components. Various anatomical and functional factors contributing to this condition are explored. This case highlights the importance of considering the Chilaiditi sign in patients with radiologic evidence of subdiaphragmatic air. It emphasizes the need for timely and accurate diagnosis to differentiate it from more severe conditions like pneumoperitoneum. Such consideration aids in optimizing management strategies and preventing unnecessary investigations.

## Introduction

Chilaiditi's sign, an infrequent occurrence with a global estimated incidence ranging from 0.025% to 0.28%, involves the interposition of the colon, typically the large intestine, between the diaphragm above and the liver below. This sign, when accompanied by abdominal pain, is termed Chilaiditi's syndrome [1]. Incidentally detected on radiological studies of the chest or abdomen, Chilaiditi's sign is a radiographic anomaly commonly confused with pneumoperitoneum. The challenge lies in clinical practice, where differentiating between pneumoperitoneum and pseudo-pneumoperitoneum on radiographs is intricate and pivotal. The distinction is vital due to substantial variations in management; urgent investigation and invasive interventions are often required for the pneumoperitoneum, whereas conservative approaches suffice for the pseudo-pneumoperitoneum [2,3]. Consequently, misidentification poses the risk of unwarranted referrals, investigations, or invasive procedures. In presenting this case, we aim to emphasize the diagnostic confusion encountered by doctors during the initial assessment of radiographic images and to delineate the disparities between the two conditions for more effective management.

## Case presentation

**Figure 1:**
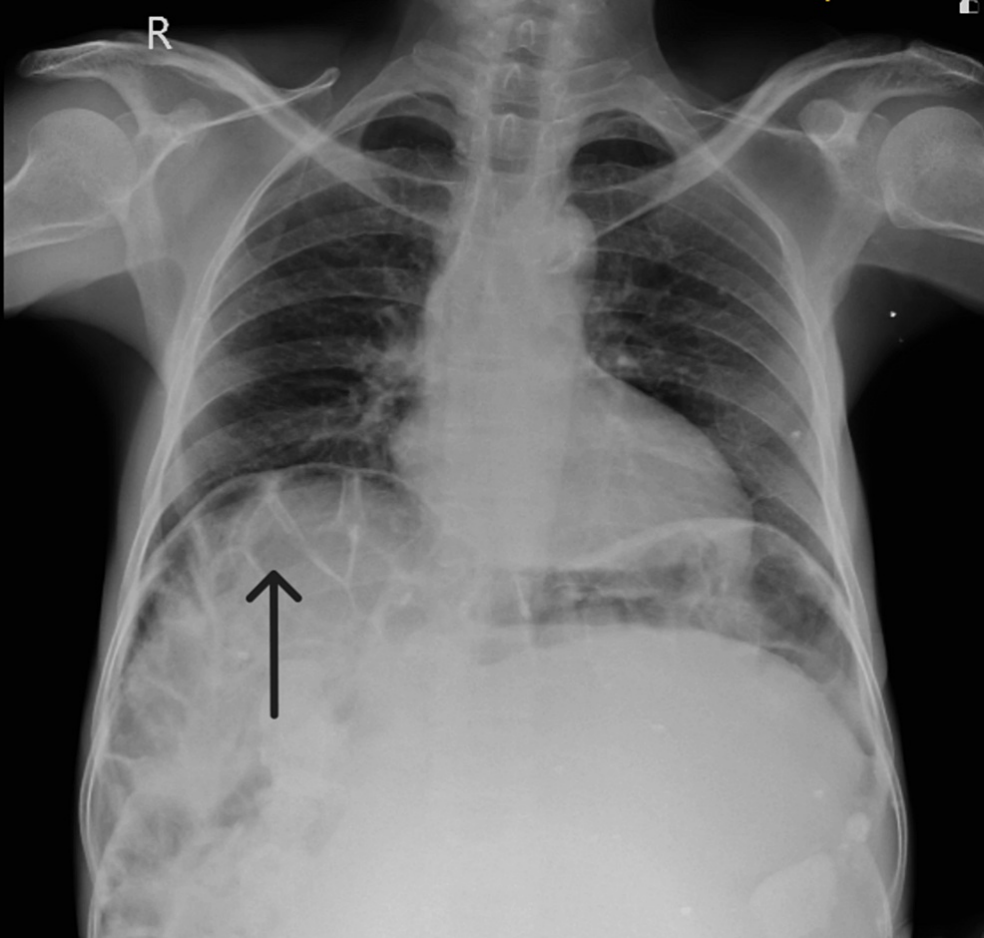
Chest X-ray (posteroanterior view) showing the elevated right hemidiaphragm with interposition of the transverse colon between the diaphragm above and liver below. Chilaiditi's sign (black arrow).

A 50-year-old woman came in the month of February of 2024 seeking medical attention at the emergency department of our hospital for diffuse on-and-off abdominal pain and breathlessness during physical activity for the past two years. Initially, the pain was intermittent and dull, localized in the lower abdomen, but it became diffuse and was accompanied by breathlessness upon exertion. Over the past two years, she had a significant weight loss of approximately 15 kg. The patient had no history of vomiting, diarrhea, or constipation. Her menstrual cycles were irregular, accompanied by dysmenorrhea, and the last menstruation was 15 days back. Upon physical examination, tachycardia was noted, with a pulse rate of 114 per minute, while her other vitals were unremarkable. Abdominal examination revealed distension, rigidity, and the presence of a solid mass upon palpation, with dullness on percussion observed in the hypochondriac, umbilical, and bilateral lumbar regions. A chest X-ray revealed an elevated right hemidiaphragm with interposition of the transverse colon between the diaphragm above and the liver below (Figure 1).

**Figure 2:**
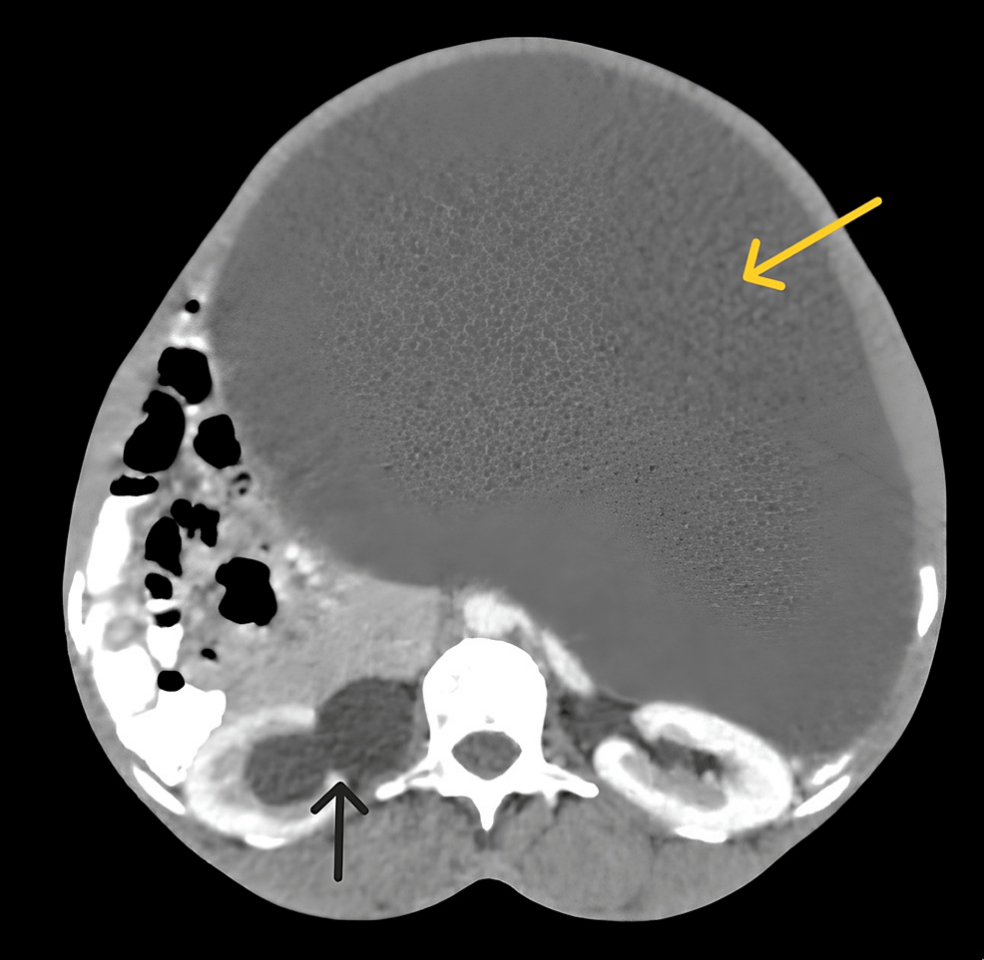
An axial section of contrast-enhanced CT scan of the abdomen showing a large cystic lobulated mass (yellow arrow) causing a mass effect on surrounding structures and hydronephrosis of the right pelvicalyceal system (black arrow).

Haustrations were observed, and gas shadow was present beneath both diaphragm domes, leading to the diagnosis of Chilaiditi's sign. To eliminate the possibility of pneumoperitoneum or any acute abdominal etiologies, the patient underwent an abdominal ultrasound promptly. The ultrasound revealed a substantial solid mass occupying nearly the entire abdominal cavity, which limited the visualization of other abdominal organs. Subsequently, a contrast-enhanced abdominal computed tomography was recommended, unveiling a large, well-defined, non-enhancing, multilobulated multicystic abdominopelvic mass lesion adherent to the anterior wall of the uterus with enhancing multiple thick internal septations and a few hyperdense areas, causing mass effects, like the compression of the right ureter leading to right-sided hydronephrosis and displacement of the bladder as well as adjacent structures (Figure 2).

These findings raised suspicion of a malignant etiology for the mass. Additionally, a heterogeneously enhancing mass in the right adnexa was identified, further heightening the suspicion of malignancy. These all investigations assisted us in ruling out the possibility of an acute pneumoperitoneum, which could have led to an emergency exploratory laparotomy surgery. After meticulous perioperative preparations, the patient was managed by gynecologists, who opted to perform a total abdominal hysterectomy, excision of the mass, and bilateral salpingo-oophorectomy for the patient. The patient tolerated the procedure well and was discharged a week later with minor postoperative weakness. The excised sample was sent for histopathological examination and the patient was asked to follow up in about seven days in the outpatient department.

## Discussion

Demetrius Chilaiditi, a radiologist, reported a case series approximately a century ago, with an accidental radiologic finding of colonic interposition between the diaphragm above and liver below, which he later coined as Chilaiditi's sign [2]. The air below the right diaphragm in radiographic pictures indicates colonic interposition, also known as the Chilaiditi sign. The following criteria must be fulfilled to identify this sign radiographically: adequate elevation of the right hemidiaphragm above the liver by the loops of the bowel, distention of the intestine with air inside indicating pseudo pneumoperitoneum, and shift of the upper liver margin below the level of the left hemidiaphragm [3].

Chilaiditi's signs and symptoms have a complex pathophysiology that involves intestinal, hepatic, and/or diaphragmatic components. In normal anatomy, suspensory ligaments and colon fixation often prevent the large intestines from being placed between the diaphragm and the liver. Some changes in normal anatomy might result in this phenomenon. These anatomical differences can be the elongation, absence, or greater flexibility of the transverse colon's suspensory ligaments and disorders such as dolichocolon or congenital malposition. Anatomic distortions can also be caused by functional illnesses that raise intra-abdominal pressures, such as chronic constipation, aerophagia, liver cirrhosis, diaphragmatic paralysis, ascites, obesity, chronic lung diseases, and numerous pregnancies. Also, psychiatric illnesses like schizophrenia and mental retardation are linked to the manifestation of the Chilaiditi sign [1,3-5].

Among the differentials for this radiographic sign, pneumoperitoneum and subphrenic abscess hold significance. Normal plicae circulares or haustral markings of the colon beneath the diaphragm can effectively exclude such severe conditions. Additionally, manipulating the patient's position with free air in the abdomen changes the position of radiolucency on X-ray, unlike a patient with a Chilaiditi sign. Also, on ultrasonography, repositioning a patient with a Chilaiditi sign does not cause a shift in the location of the gas echo, setting it apart from a patient with a pneumoperitoneum [6]. A recommended course of action when radiography or ultrasound fails to distinguish between free or intraluminal subdiaphragmatic air is to conduct a computed tomography scan of the abdomen for an accurate diagnosis, provided the patient is clinically stable [7].

Management of Chilaiditi’s sign and syndrome is usually conservative, with only a few emergency cases requiring surgical interventions like intestinal obstruction, ischemia, perforation, and volvulus. If the causative agent is a drug or psychotropic drug, abstinence from such agents may lead to the resolution of the symptoms. Some patients have also been managed with laxatives, enema, nasogastric decompression, and appropriate hydration. Surgical options in use are laparoscopic, robotic, or even open surgeries, which involve resection of the culprit colon segment, colopexy, or hepatopexy [8-10].

## Conclusions

While the Chilaiditi sign is uncommon, clinicians must consider this diagnosis when encountering patients with abdominal and/or respiratory symptoms who also exhibit a radiologic observation of air beneath the right diaphragm. In our case, we could rule out the confusion between the pneumoperitoneum and the Chilaiditi sign by performing an ultrasound examination and a contrast CT scan of the abdomen. This rare condition should be considered in the diagnostic process for individuals presenting with such symptoms and radiographic findings. This would prove beneficial in discerning it from a more severe condition such as pneumoperitoneum, thereby optimizing time efficiency and preventing unwarranted investigations, ultimately guiding the implementation of an accurate management course.
